# Diverse Epitope Specificity, Immunodominance Hierarchy, and Functional Avidity of Effector CD4 T Cells Established During Priming Is Maintained in Lung After Influenza A Virus Infection

**DOI:** 10.3389/fimmu.2018.00655

**Published:** 2018-04-06

**Authors:** Katherine A. Richards, Anthony T. DiPiazza, Ajitanuj Rattan, Zackery A. G. Knowlden, Hongmei Yang, Andrea J. Sant

**Affiliations:** ^1^David H. Smith Center for Vaccine Biology and Immunology, Department of Microbiology and Immunology, University of Rochester Medical Center, Rochester, NY, United States; ^2^Department of Biostatistics and Computational Biology, University of Rochester, Rochester, NY, United States; ^3^Viral Pathogenesis Laboratory, Vaccine Research Center NIAID, Bethesda, MD, United States

**Keywords:** CD4 T cells, influenza, lung, immunodominance, epitopes, antigen presenting cells, allophycoerythrin-conjugated

## Abstract

One of the major contributions to protective immunity to influenza viruses that is provided by virus-specific CD4 T cells is delivery of effector function to the infected lung. However, there is little known about the selection and breadth of viral epitope-specific CD4 T cells that home to the lung after their initial priming. In this study, using a mouse model of influenza A infection and an unbiased method of epitope identification, the viral epitope-specific CD4 T cells elicited after infection were identified and quantified. We found that a very diverse specificity of CD4 T cells is primed by infection, including epitopes from hemagglutinin, neuraminidase, matrix protein, nucleoprotein, and non-structural protein-1. Using peptide-specific cytokine EliSpots, the diversity and immunodominance hierarchies established in the lung-draining lymph node were compared with specificities of CD4 T cells that home to the lung. Our studies revealed that CD4 T cells of all epitope specificities identified in peripheral lymphoid tissue home back to the lung and that most of these lung-homing cells are localized within the tissue rather than the pulmonary vasculature. There is a striking shift of CD4 T cell functionality that enriches for IFN-γ production as cells are primed in the lymph node, enter the lung vasculature, and finally establish residency in the tissue, but with no apparent shifts in their functional avidity. We conclude that CD4 T cells of broad viral epitope specificity are recruited into the lung after influenza infection, where they then have the opportunity to encounter infected or antigen-bearing antigen-presenting cells.

## Introduction

The fate and function of CD4 T cells elicited by infection with influenza virus, although extensively studied by many groups [reviewed in Ref. (1–8)], are still incompletely understood. It is clear that some cells remain in the draining lymph node (dLN) and participate in the germinal center response that is needed to elicit high affinity protective antibodies. CD4 T cells that remain in the dLN can also provide help to virus-specific CD8 T cells, promoting their expansion as effector cells as well as their establishment and maintenance as memory cells. Other subsets of virus-specific CD4 T cells enter the recirculating pool and can be detected in peripheral blood and in the spleen [reviewed in Ref. (6–8)]. Finally, a major subset of virus infection primed CD4 T cells traffic to the lung where they can convey a number of effector functions including production of anti-viral cytokines such as IFN-γ, facilitation in the localization of infiltrating CD8 T cells in the airway (9), or direct cytotoxicity of antigen-bearing cells [reviewed in Ref. (10)].

Coincident with the diverse functions of CD4 T cells in immunity to influenza is their very broad epitope specificity. Studies in our laboratory using several different mouse strains have indicated that influenza infection elicits a broad array of distinct epitope specificities (~20 to >60, depending on the mouse strain) that are derived from many different influenza proteins (11–13). One question that has not yet been addressed is whether these diverse peptide specificities that are elicited by infection persist in the CD4 T cells that home to the lung after infection. For the most part, CD4 T cell trafficking to the lung after influenza infection has been studied either by using T cell receptor (TcR) transgenic CD4 T cells (14) or other methods such as tetramer staining that tracks single epitope specificities (15, 16) or by analyzing infiltrating CD4 T cells independently of their specificity (17).

We speculated that there might be several mechanisms that could contribute to restricted epitope specificity in the lung during respiratory infection relative to the dLN where priming occurs. First, recent data (18–20) suggest that the “strength of signal” delivered through the TcR can determine CD4 T cell fate between follicular helper cells (Tfh) and other types of effector cells, leading to the possibility that epitope-specific preferences might emerge soon after priming in the dLN. In fact, our laboratory recently showed that divergence between Tfh and non-Tfh cells quantified in CD4 T cells isolated from the dLN at the peak of the response differs among distinct peptide epitopes. These epitope-dependent preferences in CD4 T cell differentiation persist independently of whether the epitopes are introduced *via* infection, protein vaccination, or in a completely heterologous protein vector (21). Second, CD4 T cells that home to the lung after infection likely have the opportunity to interact with viral antigen bearing, class II positive antigen-presenting cells (APC) in the lung that are distinct from those in the lymph node (22, 23). Third, in the lung, glycoprotein viral antigen or influenza virion handling by lectin receptors (24, 25) or viral antigen abundance could lead to distinct virus epitope display than that presented in the dLN. All of these events could affect CD4 T cell specificity, effector function, as well as selectivity of the repertoire established in the CD4 T cell memory pool.

Because of the importance of this issue, in this study, we sought to empirically examine the CD4 T cell peptide specificity, drawn from the endogenous, polyclonal CD4 T cell repertoire that homes to the lung after influenza infection. Using a mouse model of influenza A H1N1 infection and an unbiased method to identify CD4 T cell epitopes elicited by influenza infection, we compared the diversity of influenza-specific CD4 T cells and immunodominance hierarchies within the lung with that established in the priming lymph node. We also examined the distribution of CD4 T cells within pulmonary vasculature and lung tissue and their cytokine potential and the avidity of their T cell receptors. Our studies revealed that most of the antigen-specific pulmonary CD4 T cells are localized to the tissue as compared to the vasculature and that the extensive degree of epitope-specific diversity observed in the dLN is maintained in the lung after infection.

## Materials and Methods

### Mice

A/JCr female mice were purchased from Charles River laboratories and were maintained in the specific pathogen-free facility at the University of Rochester according to the institutional guidelines. Mice were used at 2–6 months of age. Experiments typically involved cells from pooled tissues from six to eight mice unless otherwise noted.

### Ethics Statement

All mice were maintained in a specific-pathogen free facility at the University of Rochester Medical Center according to the institutional guidelines. All animal protocols used in this study adhere to the AAALAC International, the Animal Welfare Act, and the PHS Guide and were approved by the University of Rochester Committee on Animal Resources, Animal Welfare Assurance Number A3291-01. The protocol under which these studies were conducted was originally approved March 4, 2006 (protocol no 2006-030) and has been reviewed and re-approved every 36 months with the most recent review and approval January 23, 2018.

### Peptides

17-mer peptides overlapping by 11 amino acids to encompass the entire sequence of the HA and NA proteins from the H1N1 strain of influenza virus A/New Caledonia/20/99, the NS1 sequence from A/New York/444/2001, and the NP and M1 from A/New York/348/2003 were obtained from BEI Resources, ATCC. The internal proteins for influenza are generally conserved between the virus strains A/New Caledonia/20/99, A/New York/348/2003 and A/New York/444/2001. The peptides were reconstituted at 10 mM in PBS, with or without added dimethyl sulfoxide, to increase solubility of hydrophobic peptides, and 1 mM dithiothreitol, for cysteine containing peptides. Working stocks (1 mM) were prepared in complete DMEM, filter sterilized and stored at −20°C, as were concentrated stocks. Table 1 provides the sequences and nomenclature for the peptides used in these studies.

**Table 1:** MHC-class II restricted epitopes (A/J).

Peptide	Peptide sequence
*HA_120_	**120** EQLSSVSSFERFEIFPK **136**
*HA_174_	**174** YPNLSKSYVNNKEKEVL **190**
*HA_215_	**215** VSVVSSHYSRRFTPEIA **231**
*HA_358_	**358** TGMVDGWYGYHHQNEQG **374**
*HA_375_	**375** SGYAADQKSTQNAINGI **391**
*HA_398_	**398** VIEKMNTQFTAVGKEFN **414**
*HA_492_	***492** MESVKNGTYDYPKYSEE **508***
*NA_279_	**279** CSCYPDTGTVMCVCRDN **295**
*NA_291_	**291** VCRDNWHGSNRPWVSFN **307**
*NA_351_	**351** YKYGNGVWIGRTKSNRL **367**
*NA_381_	**381** TDTDSDFSVKQDVVAIT **397**
*NA_405_	**405** SFVQHPELTGLDCIRP **420**
*NA_416_	**416** DCIRPCFWVELVRGLPR **432**
NA_422_	**422** FWVELVRGLPRENTTIW **438**
*NA_446_	**446** FCGVNSDTANWSWPDGA **462**
*NA_452_	**452** DTANWSWPDGAELPFTI **468**
NP_49_	**49** LNDYEGRLIQNSLTIER **65**
*NP_103_	**103** KWVRELVLYDKEEIRRI **119**
*NP_127_	**127** DDATAGLTHIMIWHSNL **143**
*NP_187_	**187** GTMVLELIRMIKRGIND **203**
*NP_258_	**258** FLARSALILRGSVAHKS **274**
*NP_342_	**342** RVSSFIRGTRVLPRGKL **358**
*NP_408_	**408** TQPTFSVQRNLPFDKTT **424**
NP_414_	**414** VQRNLPFDKTTIMAAFT **430**
*NP_426_	**426** MAAFTGNTEGRTSDMRA **442**
*NP_432_	**432** NTEGRTSDMRAEIIKMM **448**
*NP_474_	**474** PIVPSFDMSNEGSYFFG **490**
*M1_37_	**37** TDLEALMEWLKTRPILS **53**
*M1_103_	**103** LKREITFHGAKEIALSY **119**
*M1_151_	**151** CEQIADSQHKSHRQMVT **167**
*M1_187_	**187** KAMEQMAGSSEQAAEAM **203**
*M1_228_	**228** GLKNDLLENLQAYQKRM **244**
NS1_13_	**13** CFLWHVRKQVADQDLGD **29**
NS1_90_	**90** LTDMTIEEMSRDWFMLM **106**
NS1_144_	**144** LTLLRAFTEEGAIVGEI **160**
NS1_174_	**174** VKNAIGVLIGGLEWNDN **190**
NS1_192_	**192** VRVSETLQRFAWRSSNE **208**
NS1_192_	**192** VRVSETLQRFAWRSSNE **208**

*Antigenic peptides from major viral proteins (HA, NA, NP, M1, and NS1) used to identify and quantify epitope specific CD4 T cells in different tissues*.

*Peptides used in pools (Figures 6 and 7) to stimulate antigen-specific CD4 T cells followed by intracellular cytokine staining.

*The bold numbers represent the amino acid numbers of the N- and C-terminal amino acids of each peptide*.

### Influenza Infections and Tissue Harvest

A/New Caledonia/20/99 virus was produced as we have previously described (11). Mice were infected intranasally at 50,000 EID_50_ in 30 μl of phosphate buffered saline (PBS) after being anesthetized by intraperitoneal injection with tribromoethanol (Avertin; 250–300 μl per mouse, normalized to weight). Spleen, mediastinal lymph nodes (mLN), and lungs were excised at 9–11 days postinfection. For cytokine EliSpots, tissues were pooled (five to eight mice) and used as the source of CD4 T cells. For some flow cytometry analyses, individual mice were sampled, as indicated in the figure legends. Lung tissue was minced and digested in cell culture media (RPMI 1640 supplemented with l-glutamine, 2.5% FBS, 10 mM HEPES) containing collagenase type II (1 mg/ml) and DNaseI (30 μg/ml) at 37°C for 30 min. Digested lung tissue and other lymphoid tissues were crushed, passed through a 40 μm mesh filter and rinsed using DMEM supplemented with 10% FBS. Samples were depleted of red blood cells (RBC) using ACK Lysis Buffer (0.15 M NH_4_Cl, 1 mM KHCO_3_, and 0.1 mM Na_2_-EDTA in H_2_O, pH 7.2). After washing, cells were depleted of B cells, CD8 T cells, and MHC class II positive cells by negative selection using MACS negative selection (Miltenyi Biotec, San Diego, CA, USA), according to the manufacturer instructions.

### EliSpot Assays

EliSpot assays were performed as previously described (12, 26, 27). Briefly, 96-well filter plates (Millipore, Billerica, MA, USA) were coated with 2 μg/ml purified rat anti-mouse IL-2 or IFNγ (clone JES6-1A12, clone AN-18, respectively, BD Biosciences, San Jose, CA, USA) in PBS overnight at 4°C, washed with media to remove any unbound antibody and incubated with 100 μl media per well for 1 h to block non-specific binding. Isolated CD4 T cells (200,000 mLN cells per well, 350,000 splenocytes per well, or 10,000 lung cells per well) were co-cultured with 500,000 syngeneic spleen cells and peptide at a final concentration of 5 μM in a total volume of 200 μl for 18–20 h at 37°C and 5% CO_2_. The cells were removed from the plates, and the plates were washed with wash buffer (1X PBS, 0.1% Tween-20). Biotinylated rat anti-mouse IL-2 or IFNγ (clone JES6-5H4, clone XMG1.2, respectively, BD Biosciences) was added at a concentration of 2 μg/ml, 50 μl/well, in wash buffer with 10% FBS and incubated at room temperature for 30 min. The plates were washed again and streptavidin-conjugated alkaline phosphatase (Jackson Immuno Research, West Grove, PA, USA) was added at a dilution of 1:1000 in wash buffer with 10% FBS, 50 μl well, and incubated for 30 min at room temperature. The plates were washed with wash buffer and developed using Vector Blue substrate kit III (Vector Laboratories, CA) prepared in 100 mM Tris, pH 8.2. After drying, quantification of spots was performed with an Immunospot reader series 5.2, using Immunospot software, version 5.1.

### Statistical Treatment of Data

Least squares linear regression models were fit to epitope-specific counts from EliSpot assays using CD4 T cells isolated from dLN and lung. Plots of residuals vs. predicted values and normal scores were examined to check the assumptions of normally distributed errors with constant variance. Studentized residuals and Cook’s distance were computed to detect potential outliers. Base library and ggplot2 package in R were used for analyses and data representation.

### T Cell Hybridoma Assays

NA446 and NP127 specific T cell hybridomas (see Table 1 for amino acid sequences and nomenclature used) were generated by fusion of TcR-negative, BW5147 lymphoma cells with peptide-activated T cells harvested from the mLN or lung isolated from New/Caledonia/20/99 virus-infected A/J mice 10 days postinfection. Selection for fused cells with HAT was followed subcloning by limiting dilution cloning such that each clone was derived from a single cell. T cell assays were performed as previously described where T cell hybridomas were co-cultured with naïve splenocytes and peptide at a range of doses (0.0001–100 μM to) for 18 h in flat bottom 96-well plates. IL-2 produced by the CD4 T cells was quantified using CTL.L and MTT assays as previously described (28).

### Intravascular Labeling and Lung Processing

Allophycoerythrin-conjugated (fAPC) anti-mouse CD45 (30-F11, Tonbo) was prepared in sterile DPBS (3 μg/mouse) and loaded into 0.5-cc insulin syringes. Mice were anesthetized using isoflurane *via* inhalation and 100 µl of antibody solution was delivered intravenously (IV) per animal via retro-orbital injection. 3 min following IV injection, blood was collected in tubes containing sodium heparin followed by intraperitoneal administration of a lethal dose of avertin. The lungs were perfused via intracardiac injection of the right ventricle with 5 ml ice-cold lung perfusion media (1× DPBS supplemented with 0.6 mM EDTA). Lung tissue was excised, dipped in HBSS and then digested as described above prior to further handling for intracellular cytokine staining (ICS) and flow cytometry. PBLs were isolated from whole blood using lymphocyte separation medium (Corning) according to the manufacturer’s instructions.

### Flow Cytometry

Single cell suspensions were stained with a viability dye, followed by incubation with purified rat anti-mouse CD16/CD32 (mouse BD Fc Block, clone 2.4G2) as previously described (22). Cells were then stained for 25 min at 4°C in the dark using antibodies targeting the following markers: CD69 (H1.2F3, Biolegend), CD4 (RM4-5, BD Biosciences), CD11a (2D7, BD Biosciences), and CD44 (IM7, Tonbo), (MEL-14, Biolegend). Cells were then washed and prepared for flow cytometry analysis. Data was acquired using a BD LSR-II instrument, configured with 488 (blue), 633 (red), 407 (violet), and 532 (green)-nm lasers. Data were analyzed using Flowjo software (FlowJo, LLC), version 10.

### Intracellular Cytokine Staining

Cells derived from infected lungs were enriched for CD4 T cells using negative paramagnetic bead selection using MACS technology. CD4 T cells were then co-cultured in u-bottom 96 well plates (3 × 10^5^ cells/well) with APC from the spleens of naïve, syngeneic donors (5 × 10^5^ cells/well) with or without exogenous, influenza peptide pools, separated by the protein from which the peptides were derived (see Table 1). A cocktail of monensin and Brefeldin A was then spiked into the cultures and incubated for 6 more hours for a total culture time of 8 h. Plates were then stored at 4°C for 12 h, cells were washed, stained with fixable live/dead Aqua, washed, and stained as described above for CD44 (IM7, Tonbo) and CD4 (RM4-5, BD Biosciences). Following incubation at 4°C for 25 min, cells were fixed and permeabilized using the eBioscience FoxP3 transcription factor staining buffer set according to manufacturer’s instructions. Intracellular proteins were then stained using the following cocktail of antibodies: IFN-γ (XMG1.2, BD Biosciences) and IL-2 (JES6-5H4, BD Biosciences), and incubated for 45 min at 4°C. Data were acquired using a BD LSR-II instrument, configured with 488 (blue), 633 (red), 407 (violet), and 532 (green)-nm lasers. Data were analyzed using Flowjo software (FlowJo, LLC), version 10. Combination Boolean gating was performed to select cytokine-responses (IFN-γ and IL-2) from single, live, antigen-experienced CD4 T cells. Frequencies for each possible cytokine pattern (2^2^) were tabulated in FlowJo, exported as an Excel file, annotated, and then finally plotted in Prism GraphPad software, version 6.

## Results

### Infection Elicits a Broad Distribution of Epitope Specific CD4 T Cells

These studies began with the goal of defining the epitope specificity in A/J (H-2^a^) mice that express the two isotypic forms of class II molecules (I-A^k^ and I-E^k^) because this offered the opportunity to sample many different epitope specificities drawn from the endogenous CD4 T cell repertoire. A peptide pooling matrix (29, 30), in conjunction with splenic CD4 T cells isolated day 9–11 postinfection, were employed in order to define the epitope specificity of CD4 T cells elicited by influenza A/New Caledonia/20/99 (H1N1) virus, as we have previously described (11–13). This method, involving cytokine EliSpot assays and overlapping peptide libraries encompassing the entire translated sequence of pathogen-derived proteins, allows an unbiased approach to identify CD4 T cells epitopes. CD4 T cells specific for five distinct viral proteins, hemagglutinin (HA), neuraminidase (NA), nucleoprotein (NP), matrix 1 (M1), and nonstructural protein 1 (NS1) were chosen for these studies (See Table 1). These viral proteins are abundantly expressed after infection and have distinct sites of localization in the virion and infected cells. Thus, these viral proteins might be handled differently *in vivo* after infection. HA and NA are transmembrane glycoproteins, M1 is associated with the cytosolic/inner membrane of infected cells/virions respectively, NP is abundant in viral ribonuclear protein complexes infected cells and virions, while NS1 is absent in virions, but is abundantly produced in infected cells and can be found in the cytosol and nucleus in infected cells (31).

**Figure 1:**
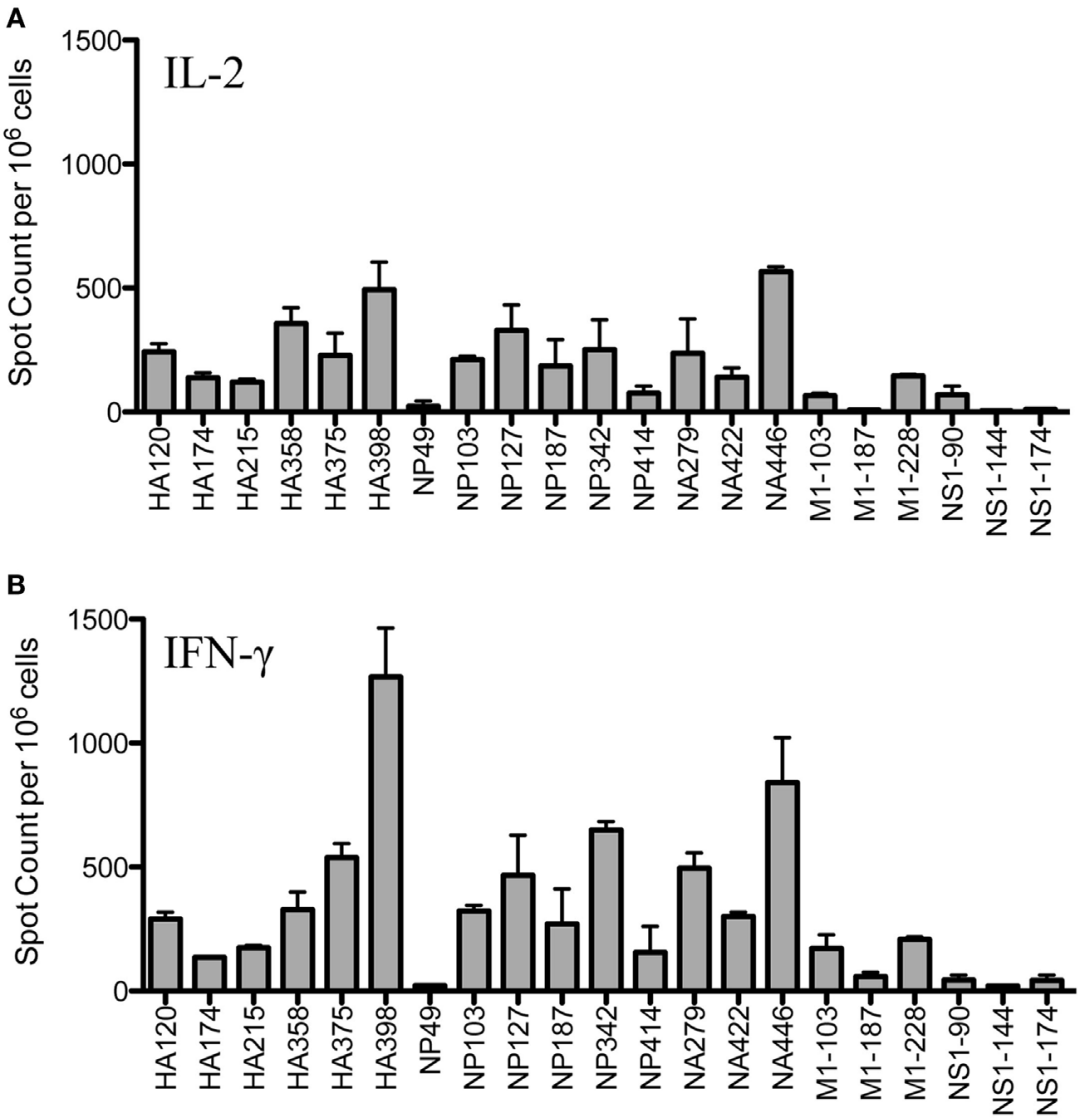
Infection elicits CD4 T cells of broad epitope diversity. Female A/J mice (aged 2–6 months) were infected intranasally with 50,000 EID_50_ of A/New Caledonia/20/99 H1N1 virus, and the number of peptide-specific cytokine producing cells from the spleen were quantified. IL-2 cytokine EliSpots **(A)** are quantified in the top panel and IFN-γ producing cells **(B)** are shown in the bottom panel. Results are represented as cytokine-producing spots per million CD4 T cells and represent the average of two independent experiments with the range indicated.

Figure 1 shows the results of replicate cytokine EliSpot assays, using IL-2 (Panel A) and IFN-γ (Panel B) to quantify peptide-reactive cells for the epitopes previously identified, including as many as is feasible, from many different influenza viral proteins. It is clear from these analyses that the epitope specificity of CD4 T cells is very broad and that CD4 T cells specific for immunodominant epitopes from HA, NP and NA could be readily identified, as well as subdominant and more minor epitope specificities from M1 and NS1. Although early studies suggested that NP and HA were the major source of epitopes recognized by CD4 T cells after infection (32), more recent studies by our laboratory have demonstrated that epitope specificity can be quite broad and depends on the selectivity imposed by MHC class II molecules (12). This analysis of the response of A/J mice after infection confirms this conclusion.

### The Broad Epitope Specificity of CD4 T Cells Detectable in the Lung dLN Is Readily Detectable in the Site of Infection

**Figure 2:**
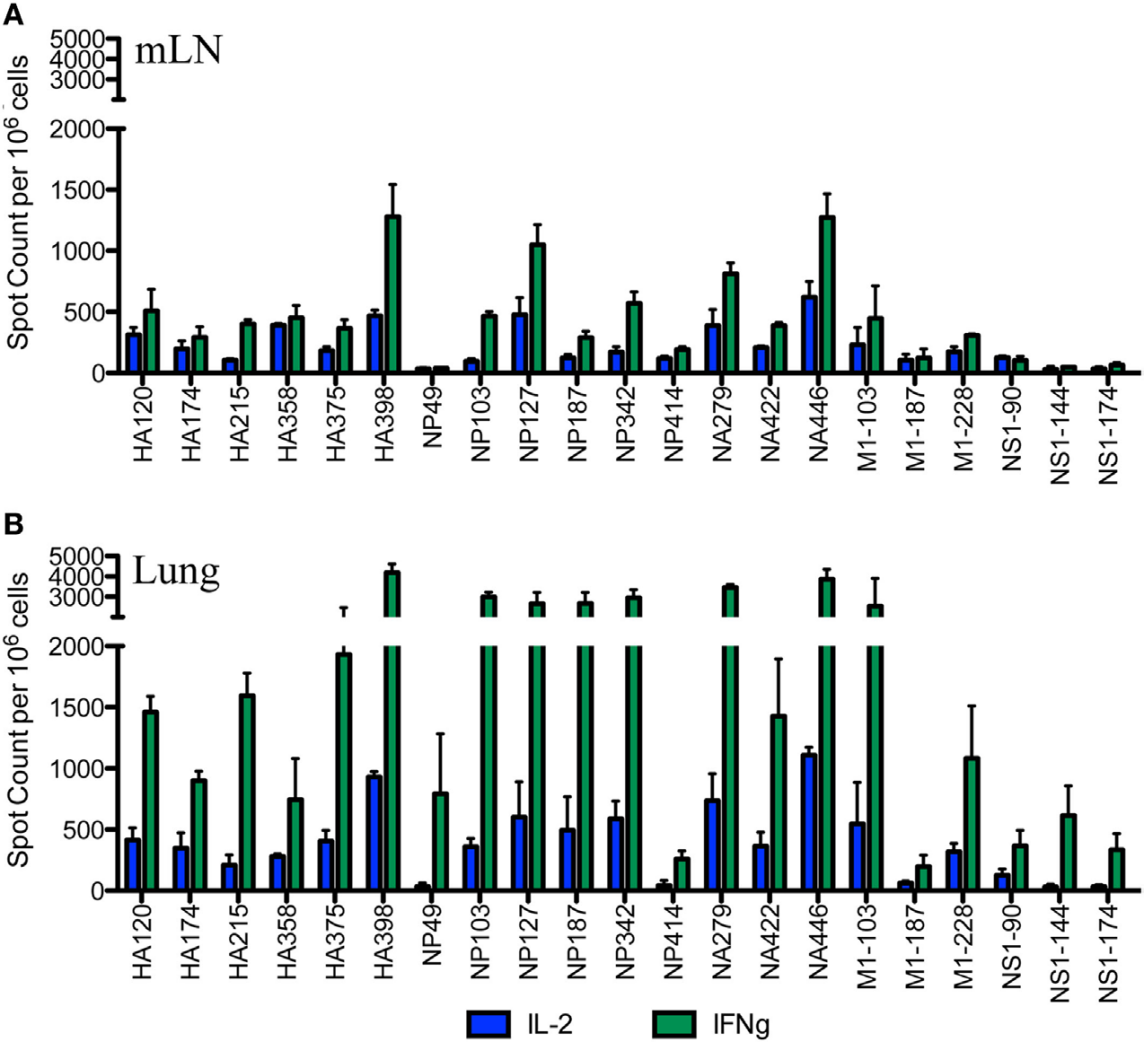
Broad CD4 T cell specificity identified in the draining lymph node (dLN) is maintained at the site of infection. Female A/J mice were infected intranasally with 50,000 EID_50_ of A/New Caledonia/20/99 H1N1 virus. The number of virus peptide-specific IL-2 producing (blue) and IFN-γ producing (green) CD4 T cells were quantified at days 10–11 postinfection in CD4 T cells using cytokine EliSpots. Cells isolated from mediastinal lymph nodes (mLN) are indicated in **(A)** and from the lung in **(B)**. Results are represented as cytokine-producing spots per million CD4 T cells and represent mean ± SD of three independent experiments.

**Figure 3:**
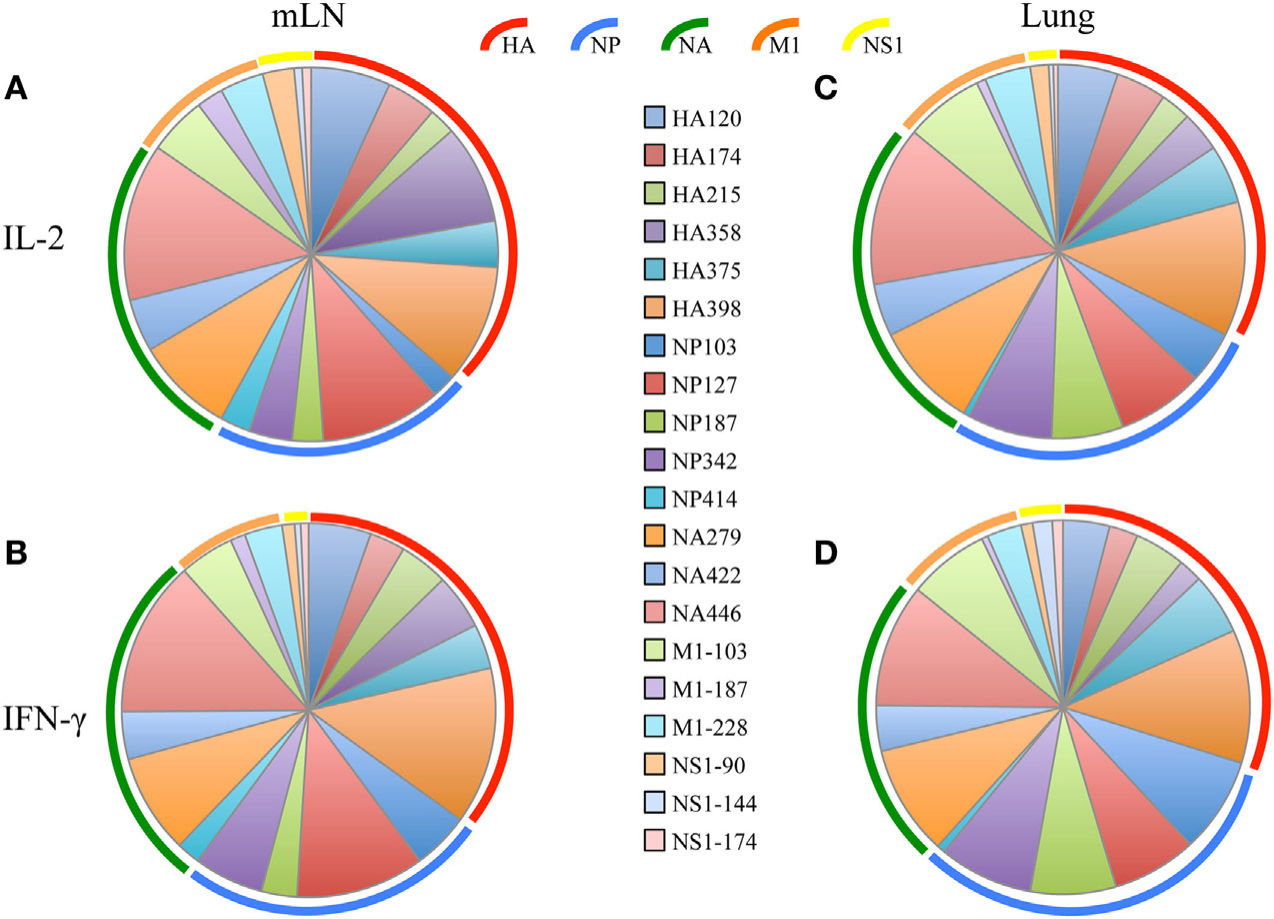
Virus-specific CD4 T cell diversity in the draining lymph node (dLN) and lung. The number of peptide specific IL-2 producing CD4 T cells was quantified at day 10–11 postinfection by cytokine ELISPOT assay as indicated in Figure 2. The average percent response recruited by each peptide was calculated by the dividing the number of cytokine EliSpots elicited by the given peptide by the sum of the EliSpot responses elicited by all of the peptides tested. This percent response value is represented as a “slice” of the pie for mediastinal lymph nodes (mLN) **(A,B)** and Lung **(C,D)** for IL-2 **(A,C)** and IFN-γ **(B,D)**. The different color arcs indicate the source protein each epitope. Each individual epitope is indicated by a different colored slice of the pie and is colored for each peptide as indicated in the center panel. Results represent the average of the three independent experiments shown in Figure 2 with the indicated SD.

After the primary response, a fraction of the responding CD4 T cells migrate to the respiratory tract. Early studies by Woodland *et al*. (32) showed that a CD4 T cell repertoire with diverse epitope specificity can be detected in the lung after infection. In the experiments here, we sought to perform side-by-side analyses of CD4 T cells present at the peak of the response in the lymph node with those cells that had migrated to the lung. The peptides validated by replicate assays shown in Figure 1 were used to sample CD4 T cells from the lung-draining mLN and lung at days 9–11 after infection. Figure 2 shows the results of these analyses, where mLN responses are shown in Panel A and lung-specific responses are shown Panel B. IL-2 (blue bars) and IFN-γ (green bars) producing cells were both sampled in peptide-stimulated cytokine EliSpots assays. It is clear from this side-by-side analysis that the CD4 T cell specificities primed in the lung-dLN can be readily detected in the lung. Both IL-2 and IFN-γ producers are detectable in the lung, although there is a striking shift in cytokine profile, with IFN-γ producing cells becoming much more prominently represented in CD4 T cells isolated from the lung relative to those from the LN (note the differences in scales used for cytokine producing cells in LN and lung). Even the minor epitope specificities, such those derived from NS1 and M1, are readily detectable in the lung. Thus, the broad peptide specificity in CD4 T cells primed in the lymph node persists in CD4 T cells isolated from the lung. We noted no striking gains or losses of CD4 T cell specificity when the dLN and lung are compared. Figure 3 illustrates the diversity in lung homing CD4 T cells in a readily visualized manner using a pie diagram. Here, every peptide specificity that was tested is denoted by a different color with the width of the pie “slice” indicating the relative abundance of the epitope specificity, using either IL-2 or IFN-γ EliSpots as an indicator of the magnitude of the response to each peptide. The protein derivation of each of the peptides sampled is indicated by the outer arc in these diagrams. These studies show that each peptide specificity detected in the LN is represented in the lung independently of its protein origin. It is clear that our estimate of the immunodominance hierarchy is dependent on cytokine production by the virus-specific CD4 T cells. Alternative measures, such as tetramer staining, potentially detect of CD4 T cells that do not produce cytokine but are limited by their ability to detect only high affinity CD4 T cells, perhaps encompassing only 5–30% of the total antigen-specific CD4 T cell repertoire (33–35).

**Figure 4:**
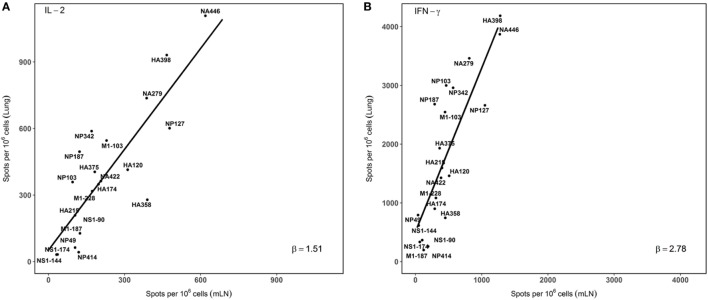
Immunodominance hierarchy of epitope specific CD4 T cells isolated from draining lymph node (dLN) is preserved in the lung lung. IL-2 and IFN-γ Elispots were performed as described from cells isolated at day 10–11 postinfection. Individual values for IL-2 **(A)** or IFN-γ **(B)** EliSpots from three independent experiments from Figure 2, with the SD values indicated in Figure 2. The average values were tabulated and analyzed using linear regression models, using each data point. The slopes of the derived line (“β”) were calculated and are indicated in each panel. Shown are the average values of spots per million CD4 T cells for each epitope data set tested. **(A)** beta (slope) = 1.51. *r*^2^ = 0.63. *p* value < 0.001. **(B)**: beta (slope) = 2.78, *r*^2^ = 0.64. *p* value < 0.001.

In order to visualize the absolute immunodominance of the peptide epitopes in the two different sites that were sampled after infection, the average number of cytokine producing cells (per million CD4 T cells) from three independent experiments was ranked in a two dimensional plot that allows the relative hierarchy to be readily visualized. These data, shown in Figure 4, indicate that epitope specificities that were dominant in the mLN, such as HA398, NP127, NA446, and NA279, remain immunodominant in the lung, while subdominant epitopes, such as HA215 and NP342, are also subdominant in the lung. Minor epitope specificities detected in the dLN, such as NS190, although detectable in the lung, remain minor. The consistent immunodominance is evidenced by the general adherence to the straight line shown in panels A and B, for IL-2 and IFN-γ, respectively. We did note several epitope specificities (e.g., M1-103 and NP187) measured by both IL-2 and IFN-γ producing cells that were somewhat enriched in the lung relative to the dLN, but do not yet understand the basis for this enrichment. Interestingly, the slopes of the lines are distinct for IL-2 (1.51) *vs*. IFN-γ (2.78) again emphasizing the accumulation of IFN-γ-producing CD4 T cells in the lung, relative to what is detected in the draining LN, where the CD4 T cells are elicited after infection.

### Compartmentalization of Cytokine-Producing Cells in the Lung following Infection

**Figure 5:**
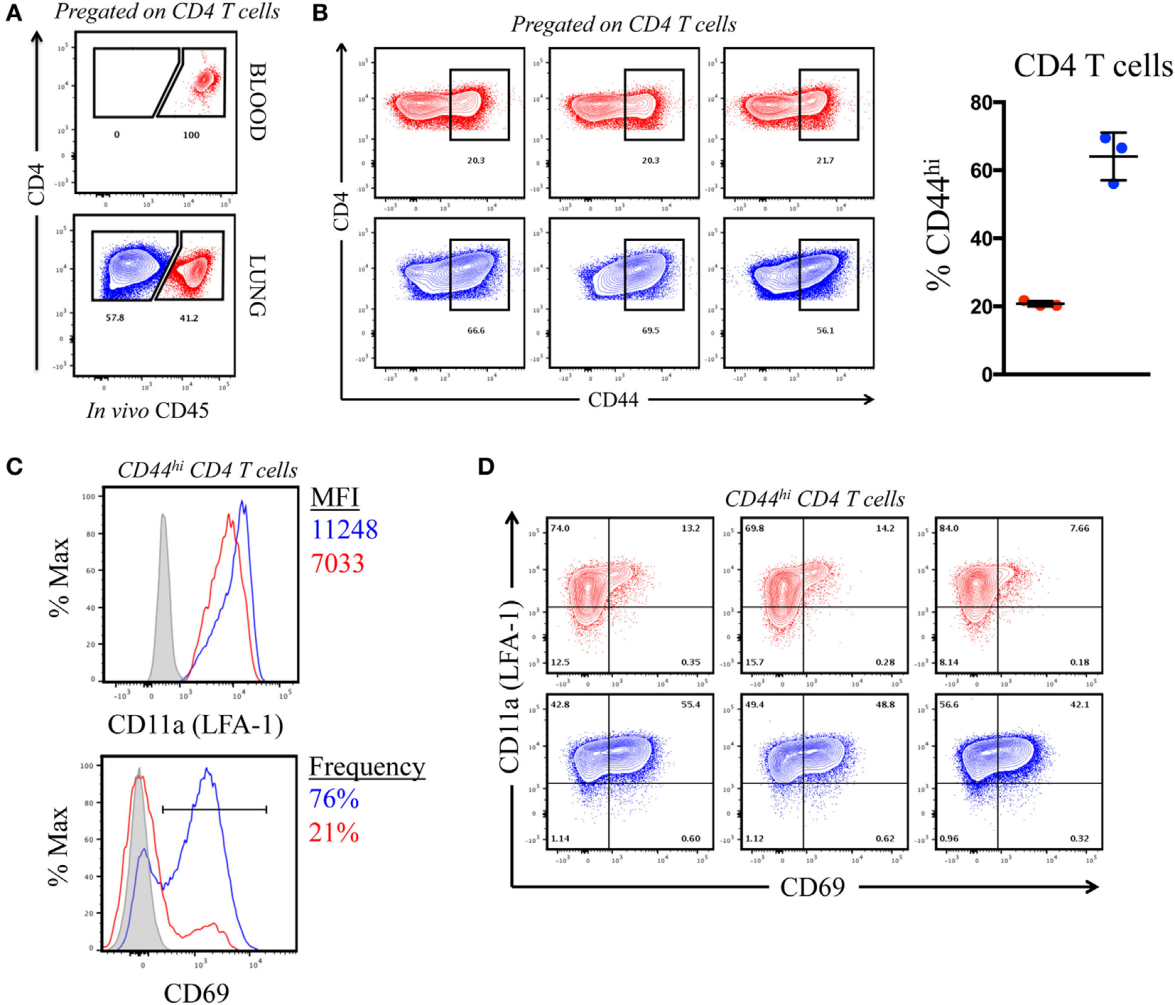
Intravenously (IV) labeling reveals distinct compartmentalization and phenotype of pulmonary CD4 T cells following infection. **(A)** Representative IV CD45-fAPC staining profile from pre-gated, CD4+ T cells in blood and lung following infection, where blue denotes “protected” cells and red denotes “labeled” cells. **(B)** Contour plots (CD4 *vs*. CD44) (pre-gated on CD4^+^) and tabulated frequency of CD44^hi^ CD4 T cells from three individual mice, shown at right. Symbols represent individual animals and error bars reflect standard deviation from the mean. **(C)** CD11a (LFA-1) MFI,% CD69^+^ (from CD44^hi^ CD4 T cells), and expression profile of CD69 *vs*. CD11a (LFA-1) on IV “labeled” and “protected” cells 10 dpi. Histograms reflect pooled responses from three animals and individual contour plots **(D)** reflect responses from the three individual animals.

The previous results, suggesting that CD4 T cells of each specificity primed in the LN home to the lung after influenza infection, were unexpected because of the distinct types of APC in the lung that may have the potential to remodel the CD4 T cell repertoire. However, it is now understood that lung-localized lymphocytes can be situated into two discrete sites following infection: the vasculature and the tissue, the latter of which includes parenchyma, interstitium, and the airways (36–39). We considered the possibility that the virus-specific CD4 T cells that were extracted from the lung and quantified by peptide-induced cytokine EliSpots were localized to the vasculature and thus had not yet encountered pulmonary APC. Cells within the lung vasculature and the tissue can be distinguished by the accessibility to antibodies that are introduced into the bloodstream shortly before animal euthanasia (40, 41). Using this method, cells in the lung vasculature become labeled, whereas cells in the tissue are “protected” from this short-term introduction of antibody. To evaluate the location of the cytokine-producing cells quantified in the previous experiments, A/J mice infected 10 days previously were injected IV with an allophycoerythrin-conjugated (fAPC)-CD45 antibody that would label all hematopoietic cells. Mice were sacrificed 3 min later and lung homogenates were prepared, and the resulting cell population was analyzed by flow cytometry. Figure 5A (top panel) shows that 100% of the peripheral blood CD4 T cells stained positive directly *ex vivo*, assuring us that the IV-introduced CD45 was sufficient to saturate circulating cells. Within the cells isolated from the lung (Figure 5A, bottom panel) approximately 40% of the total CD4 T cells were accessible to the IV antibody (pseudocolored in red in this and future data sets), while a substantial proportion (60%) of the CD4 T cells were protected from labeling (pseudocolored in blue). We next evaluated the phenotype of the CD4 T cells distinguished by CD45 staining, using the markers associated with antigen experience (CD44) and with those associated with tissue residence [CD69 and CD11a (LFA-1)] (38, 42, 43). Figure 5B shows that the CD4 T cells in the vasculature were primarily composed of naïve cells, whereas cells in the protected tissue were greatly enriched for antigen experienced, CD44^hi^ cells. Figure 5C shows a characterization of cells for the markers CD11a and CD69. The integrin CD11a, important in T cell activation and for extravasation into tissue sites, has been shown to have increased cell surface density on tissue-localized cells, while CD69, a prototypical marker of tissue-resident T cells is known to enhance tissue localization in part through its reciprocal antagonism of the sphingosine 1 phosphate receptor (36, 44). Figure 5C shows the co-expression of these markers on CD44^hi^ cells in the vasculature (in red) vs. the protected tissue (in blue). Among the CD44 bright cells, CD4 T cells in the tissue show increased cell surface density of CD11a (MFI = 11,000) compared to vascular localized (MFI = 7,000) and are greatly enriched for CD69 expression (76% vs. 21%, respectively). The data from individual animals, shown in Figure 5D, illustrate the reproducibility in the markers expressed on these distinct lung subsets. We conclude from these data that the IV labeling successfully distinguished CD4 T cells in the lung vasculature vs. the lung tissue, and that in this system, IV-protected cells have the markers associated with residence in the inflammatory microenvironment in the lung and thus the potential to contact a diverse set of lung-localized APC within the infected lung. However, this selective lung homing behavior and residency does not appear to restrict epitope specificity.

### Viral Antigen-Specific Cytokine-Producing Cells Are Primarily Contained within the Lung Tissue

**Figure 6:**
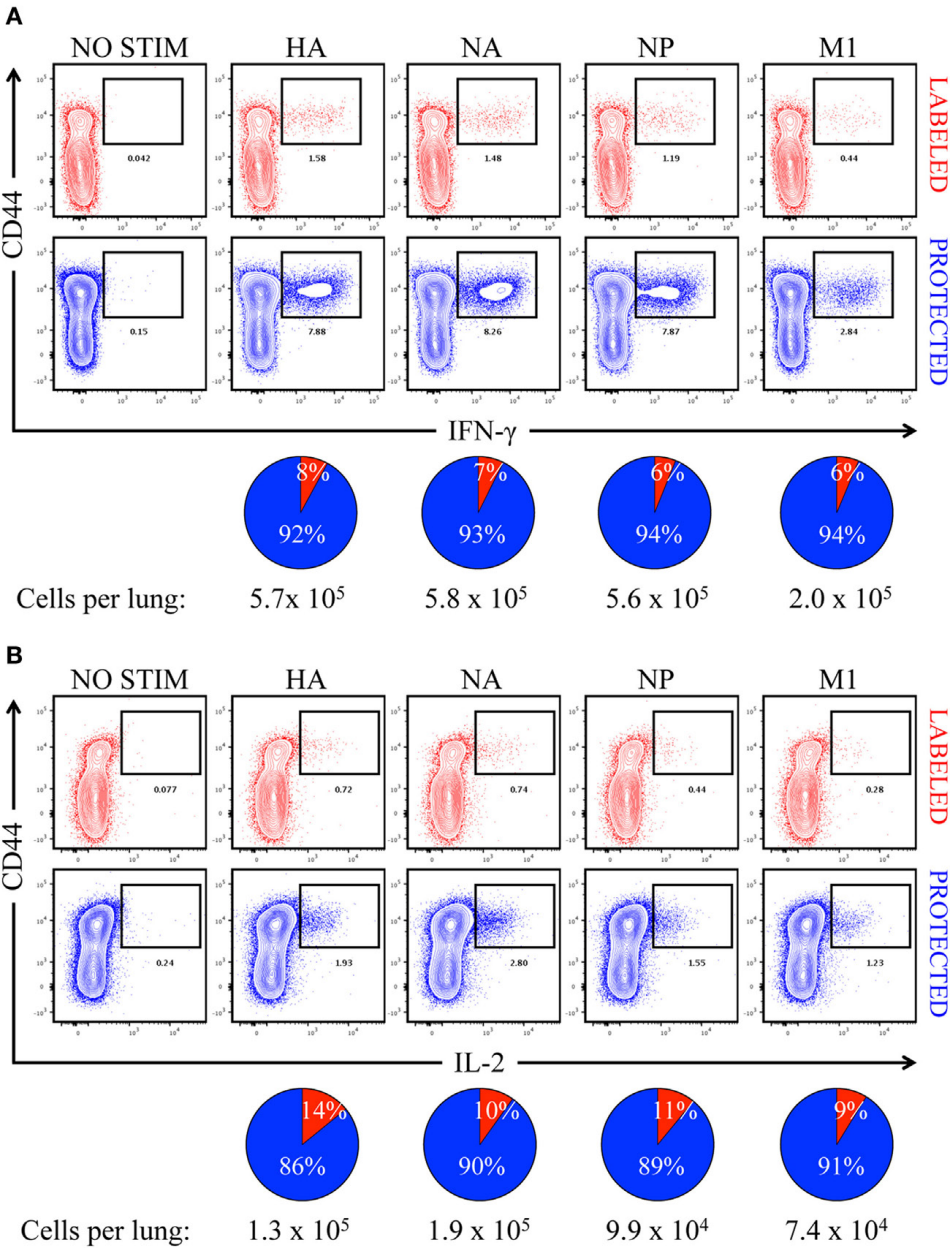
Virus-specific, cytokine-producing CD4 T cells are enriched in “protected” niches in the lung following influenza virus infection. Contour plots illustrating antigen-specific reactivity to HA, NA, NP, and M1 proteins by intravenously (IV) “labeled” (red) and “protected” (blue) pulmonary CD44^hi^ CD4 T cells, as measured by **(A)** IFN-γ and **(B)** IL-2 production. Pools of peptides from each of four different viral proteins, indicated above each panel, were used for stimulation. The pie charts below each cytokine set reflect the fraction of cytokine-producing CD4 T cells in pulmonary vasculature *vs*. tissue (vasculature in red and tissue in blue), with the total number of responding cells indicated. The responses shown are from pooled samples from five animals.

In order to determine the primary location of lung CD4 T cells whose epitope specificity was determined by cytokine EliSpots shown in Figure 2, mice were infected and sampled after infection after a brief IV treatment with fAPC-CD45 antibody. Peptide-induced ICS was then performed from the lung-isolated cells in conjunction with the fAPC-CD45 staining to distinguish CD4 T cells in vasculature and tissue. Peptide epitopes derived from the major viral proteins that serve as the source antigen (HA, NA, NP, and M1) were pooled based on their protein origin and co-cultured for 8 h with the cells isolated from the lung. Production of IFN-γ (Figure 6A) and IL-2 (Figure 6B) by the CD4 T cells was quantified by ICS and flow cytometry, using CD44 cell surface density to identify antigen-experienced cells. In these figures, IV-labeled, vasculature-localized cells are indicated (in red) in the top panels and IV-protected tissue cells (indicated in blue) are shown in the bottom panels. The percentage of total cytokine-producing cells is indicated in the pie graphs below each flow cytometry profile. The results from these analyses indicated that 92–94% of the virus-specific IFN-γ production was produced by cells in the lung tissue, while 86–90% of the IL-2 producing cells were within the lung tissue. There was no notable difference in the distribution of cytokine-producing cells specific for different viral proteins. We conclude from these analyses that the vast majority of cells assayed by cytokine EliSpots were from CD4 T cells that had entered the lung tissue and do not simply represent circulating CD4 T cells trapped in the lung vasculature. These infiltrating, virus-specific CD4 T cells thus had an opportunity to survey any viral peptide-bearing APC within the tissue.

**Figure 7:**
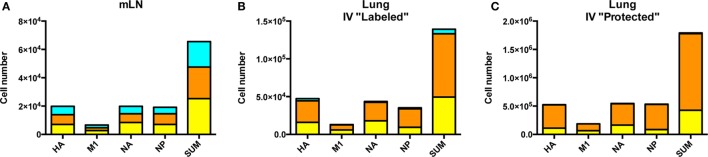
Cytokine pattern of virus-specific CD4 T cells is distinct in lymph node and lung. Cytokine profile of antigen-reactive CD4 T cells in the draining lymph node (dLN) **(A)**, lung vasculature, **(B)** and lung tissue **(C)** identified by protein specificity. Cells producing IL-2 only are represented in turquoise, IFN-γ alone indicated in orange and cells producing both IL-2 and IFN-γ are indicated in yellow. Data reflects responses from samples pooled from five animals. Note the differences in scales for CD4 T cells from each tissue.

Comparison of the cytokine profiles within the cells localized to dLN, pulmonary vasculature and lung tissue were then analyzed in more detail (Figure 7). CD4 T cells specific for each protein-specific pool of peptides were quantified for IL-2 and IFN-γ and co-cytokine production by sequential gating strategies. Figure 7A shows the cytokine polyfunctionality in the dLN, where the cells are distributed almost equally between cells that produce only one cytokine: IL-2 only (turquoise), IFN-γ only (orange) and cells that produce both cytokines IFN-γ^+^, IL-2^+^ (yellow). When the cells in the vasculature are analyzed (Figure 7B), there is an apparent commitment of lung homing cells to a Th1 IFN-γ producing population, with only a minor fraction of cells that exclusively produce IL-2. In the lung tissue (Figure 7C), the virus-specific cells are comprised primarily of IFN-γ producing cells, with most of the CD4 T cells producing only IFN-γ and very few cells producing only IL-2. Thus, there appears to be a readily detectable and progressive enrichment of cells toward IFN-γ producers from the site of CD4 T cell priming in the dLN, to when the cells traffic *via* circulation to the lung vasculature and finally establish residency in the lung tissue. All viral protein specificities appear to progress from the most diverse in cytokine potential within the lymph node, toward the most restricted cytokine-producing cells within the lung tissue. The vasculature-localized cells are intermediate between these extremes, containing some virus-specific cells producing only IL-2, a phenotype that is very poorly represented in the lung tissue. Overall, these data are consistent with the priming of CD4 T cells with diverse functional potential and for selection of a subset of these virus-specific CD4 T cells, enriched for IFN-γ potential for traffic to the lung after influenza infection.

### Functional Avidity of Epitope-Specific CD4 T Cells Isolated From mLN and Lung

We next considered the possibility that, although the immunodominance hierarchy of CD4 T cells had not been altered from that in the LN to those that ultimately establish residence in the lung, the affinity of the TcR for its ligand may be distinct in CD4 T cells from the two compartments. There has been a number of recent studies suggesting that TcR signal strength, largely determined by the affinity and/or off rate of the TcR to its ligand, can influence the fate of CD4 T cells after priming (18–20). We sought to assess the functional avidity of virus-specific CD4 T cells isolated from lung vs. the dLN at the peak of the adaptive response, independently of any potential differences in signaling capacity or expression of co-stimulatory molecules, which can affect sensitivity to antigen or cytokine responses (45–47). Therefore, we established virus-specific CD4 T cell hybridomas. CD4 T cells isolated from the draining LN and lung were restimulated with antigenic peptides, and then T cell blasts were fused to the TcR-negative T cell lymphoma BW5147. Using the TcR negative BW5147 cell line as the fusion partner, the only source of T cell receptors are those expressed by the CD4 T cells from the individual cell which has entered into a fusion with the BW5147, thus allowing us to directly query the receptors expressed on the CD4 T cells from the host (48, 49). After selection, CD4 T cell hybridomas were derived and identified. This immortalization allows the T cell receptors to now be expressed on a “neutral” cellular background, allowing more objective comparative analyses of their relative avidity for antigen, thus will exclude the properties of freshly isolated CD4 T cells whose differentiation state or expression of co-stimulatory molecules can affect sensitivity to antigen.

**Figure 8:**
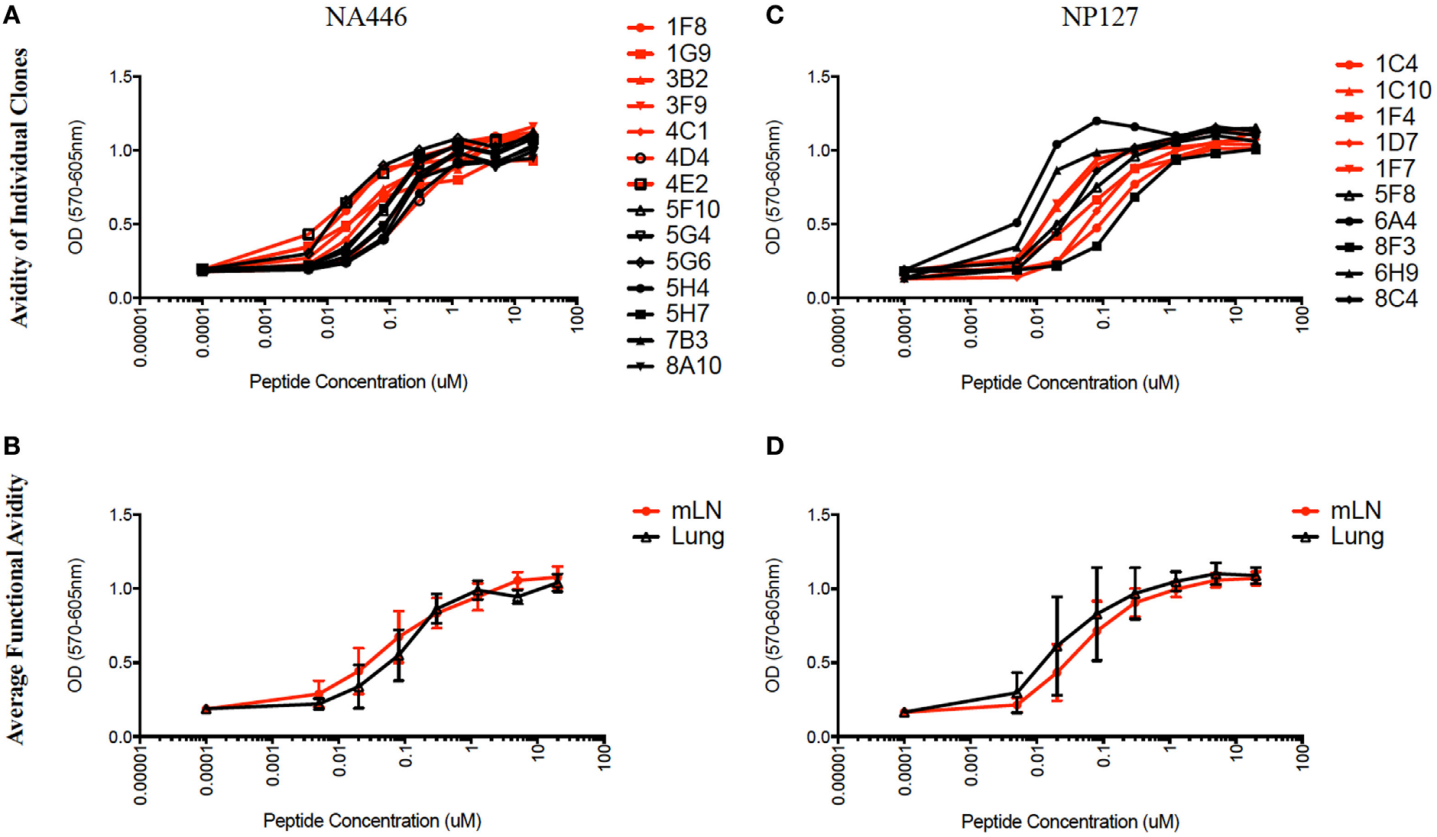
Similar range in functional TcR avidity from CD4 T cells isolated from dLN and lung. NA (NA446) and NP (NP127) specific T cell hybridomas, generated from CD4 T cells isolated from the mLN and lungs of infected mice at day 10 postinfection were assayed for their ability to produce IL-2 in EliSpot when restimulated with the indicated antigenic peptides at a range of doses (0.0001–100 μM). **(A)** The left top panel shows the responses of clones specific for NA446 from the lymph node (black lines) and lung (red lines) and **(C)** the top right panel shows the response of clones specific for NP127 from the lymph node (black) and lung (red). **(B,D)** The bottom panels illustrate the average dose–response curves of the clones. IL-2 produced by the T cell hybridomas was quantified using CTL.L and MTT assays.

The functional avidity of isolated T cell hybridoma clones that express distinct TcR V beta segments for two dominant epitopes were then compared using peptide dose-response curves, where TcR engagement was quantified by IL-2 production, which is the cytokine routinely used to assay T cell hybridomas. We (50) and others (51–54) have shown that hybridoma cells provide a very sensitive measure of functional TcR avidity. The results of these experiments are shown in Figure 8 and Table 2. Individual clones of CD4 T cell hybridomas specific for NA446 (left) or NP127 (right) from each tissue were tested in peptide dose-response assays, and IL-2 was quantified using CTLL, an IL-2 dependent cell line. The peptide dose-response curves are shown from the hybridomas isolated from dLN (in red) or from lung (in black) are shown the top panels, with the average values shown in the bottom panels. Table 2 provides that half maximal dose of peptide needed to activate the individual clones. This variability in functional avidity, as well as our studies indicating that many clones express distinct TcR Vbeta segments (not shown), indicates that the clones of hybridomas are not replicates of each other. Although there were notable differences in sensitivity to antigenic peptide by the individual CD4 T cell hybridomas, the ranges in “functional avidity” between CD4 T cells derived from dLN and lung were indistinguishable. We conclude from this that the CD4 T cell repertoire of T cells that home to the lung is not dramatically remodeled with regard to the functional avidities of the CD4 T cells isolated from the lung dLN.

**Table 2:** Relative affinities of T cell hybridoma clones.

Specificity	Tissue			
	mLN		Lung	
	Hybridoma	50% max concentration (μM)	Hybridoma	50% max concentration (μM)
NA446	4E2	0.02	5G6	0.025
	1F8	0.025	5F10	0.1
	3B2	0.055	5H7	0.1
	4C1	0.09	5G4	0.18
	1G9	0.1	7B3	0.2
	3F9	0.2	8A10	0.2
	4D4	0.4	5H4	0.3

NP127	1F7	0.02	6A4	0.0085
	1C10	0.025	6H9	0.015
	1F4	0.09	8C4	0.045
	1D7	0.15	5F8	0.07
	1C4	0.2	8F3	0.4

## Discussion

It is now clear that influenza-specific CD4 T cells have many diverse functions in the lung, some of which, such as cytotoxicity and cytokine production, likely involve contact with APC [reviewed in Ref. (55)]. In some systems, lung-homing CD4 T cells can also help in tissue-localized B cell responses (1, 56). In this study, we have evaluated whether the diverse CD4 T cell epitope specificity that is elicited in the lung dLN after influenza infection persists in the CD4 T cells that home to the lung. We were interested in this issue because recent data suggest that CD4 T cell fate can be influenced by the specific features of the peptide:class II-T cell receptor complex (18–20). These recent studies, coupled with our increasing appreciation of the multiplicity of functions that CD4 T cells carry out in the lung (42, 57) and the distinctive antigen bearing cells within the lung after infection (22, 58–60), raised the possibility that the CD4 T cell immunodominance hierarchy established in the dLN after infection might be remodeled as CD4 T cells are selected for lung homing during priming or alternatively, after establishment of residency in the lung tissue. Our studies revealed that when assayed by epitope-specific cytokine production, the epitope specificity of influenza-specific CD4 T cells is very broad in the dLN and that this diverse peptide specificity persists in cells that home to the lung. Also, the immunodominance hierarchies established in the dLN are maintained in lung CD4 T cells.

The factors that control CD4 T cell homing to the lung after influenza infection are not well defined. Recent studies have implicated upregulation of chemokine receptors such as CXCR3 (61) and CCR4 (62) during priming as an important event in homing and establishing residency in the lung after respiratory pathogen infection. Upregulation of chemokine receptors such as CXCR3 has been observed with Th1 priming in the lymph node after infection with some respiratory pathogens (63–65) and has been suggested to play a role into recruitment in the lung after intranasal infection or vaccination (62). In the infection model studied here, we also observed a strong enrichment for IFN-γ-producing cells in the lung, for all epitopes examined, a result consistent with the Th1 CD4 T cell phenotype typical of influenza CD4 T cell immunity. Enrichment for IFN-γ in the cells that traffic to the lung suggests that the early priming events in the dLN delivered signals to the elicited CD4 T cells that promoted lung homing and IFN-γ production, features that appear to be functionally linked in most respiratory virus responses. The nature of the APC that are involved in initial of priming CD4 T cells in responses to infection or vaccination is not well understood, but may involve multiple subsets contacted sequentially (64, 66). The expression of co-factors that control trafficking of MHC class II, proteolysis of antigen, and editing of the final displayed epitopes on different APC (67, 68) have not been characterized in responses to respiratory infection. If there are specialized APC that promote homing of the responding CD4 T cells to the lung, our data suggest that these APC present the full diversity of virus-derived peptides, permitting all of the CD4 T cell specificities primed to be included in the subset of CD4 T cells destined for the lung. As of yet, there is little known about the TcR-mediated signaling events that might preferentially poise cells during priming of the naïve CD4 T cell repertoire to be selected to the CXCR3/Th1 pathway of differentiation that determines lung homing potential. Recent studies suggest that choices in the fate of CD4 T cells after priming (e.g., Tfh *vs*. non-Tfh “effector cells”) can be strongly influenced by specific features of peptide:class II-TcR complexes described by their “signal strength” or functional avidity [reviewed in Ref. (19)]. However, the data accumulated thus far have sometimes been contradictory and, in any case, have not yet analyzed the priming events or APC that poise CD4 T cells elicited by respiratory infection to leave the dLN and migrate to the lung. The paucity of information is particularly notable for polyclonal CD4 T cells drawn from the endogenous response. In the studies reported here, when the TcR repertoire for CD4 T cells specific for two dominant influenza epitopes isolated from dLN and lung were examined here for “functional avidity” using peptide dose–response curves, although there were a range in sensitivity to antigenic peptides among individual clones, the range in the avidities was indistinguishable within CD4 T cells isolated from the two compartments, suggesting that CD4 cells selected for homing to the tissue were not atypical with regard to affinity from the range elicited during priming and that persist in the dLN at day 10–11 postinfection. Although it would be of interest to study the CD4 T cell repertoire at later time points, as immune memory is established, several factors preclude analyses of this for the purposes of this study. First, in a comprehensive survey of the immunodominance in CD4 T cell responses to influenza using the HLA-DR1 mouse strain, we found that the abundance of total influenza reactive CD4 T cells in the periphery at memory time points (e.g., day 30–60 postinfection) diminished to 5% of the levels detected at the peak of the response (day 8–10 postinfection). In this earlier study, we tracked more than 50 different influenza epitopes. Remarkably, although much lower in abundance, the CD4 T cell immunodominance hierarchy was stable (69). For the current study, when we have examined the abundance and distribution of CD4 T cells at an even modestly extended time (day 18 postinfection), the number of antigen-experienced CD4 T cells in the lung drops dramatically, to less than 15% of that seen at the peak. Even more important, using the IV labeling technique, we found that the total lung CD4 T cells are now composed equally of vasculature and tissue localized cells (data not shown). Because in the current study, we explicitly sought to examine cells within the unique microenvironment of the lung tissue after infection, analyzing CD4 T cell specificity at even just a few additional weeks postinfection would be complicated by the very large representation of CD4 cells from the lung vasculature.

The persistence in broad immunodominance, with little skewing or narrowing of specificity or functional avidity, raised the possibility that the cells detected from disrupted lung tissue were contained within the vasculature and had not yet entered the infected lung and contacted lung APC. However, our studies revealed that most of the cytokine-producing cells within the lung were within the lung tissue, rather than the vasculature, suggesting that the infiltrating cells likely have had the opportunity to encounter potential antigen bearing cells in the lung. There have been several recent studies that have suggested that viral antigens persist long after virus is cleared from the lungs (70, 71). Early studies included the demonstration that viral peptide:MHC class II complexes at levels sufficient to activate adoptively transferred CD4 T cells persist in the lung draining LN for at least 60 days postinfection (70). More recent studies indicated that viral RNA and viral protein can be detected primarily in radio-resistant non-hematopoietic cells in the lung tissue for more than 2 weeks after virus is cleared from the lung (70). Finally, contact of infiltrating T cells with lung APC after infection has been supported by recent reports in the literature (55, 72–76).

Whether influenza viral antigens are accessible in the form of peptide:class II complexes to infiltrating CD4 T cells and persists as the adaptive response progresses in the infection model studied here is not known. For influenza-specific MHC class I restricted T cells, Legge and coworkers provided evidence that recruited dendritic cells that persist in the lung serve as APC for newly primed infiltrating virus-specific CD8 T cells (74). Recent studies by Swain *et al*. of CD4 T cells primed in influenza infection suggest that late (e.g., day 5–7 postinfection) contact with antigen bearing cells is important in establishing long lived virus-specific CD4 T cell memory. In this study, tracking of epitope-specific cells and use of reporters that detect TcR engagement suggested that these later (day 5–7 postinfection) antigen-dependent interactions occur in the lung (77). Collectively, these studies suggest that influenza-specific CD4 T cells that home to the lung after infection can encounter peptide:MHC class II complexes and such contact might affect survival and potential remodeling of the immunodominance hierarchy. Our recent studies performed early after infection with a Venus reporter virus suggests that a diverse array of both bone marrow derived and non-bone marrow derived, class II positive cells acquire viral proteins during infection and that many of these subsets express detectable viral peptide:class II complex (22). However, the anatomic distribution and persistence of these diverse antigen-bearing APC are not known at this time, nor their options for encounter with lung-infiltrating CD4 T cells after primary infection. We have not yet quantified the epitope display on MHC class II positive cells in the lung at later time points when CD4 T cells begin to infiltrate the lung (day 5–6 postinfection) or the presence long lived antigen depots in this infection system. Thus, we do not yet know whether the lung homing CD4 T cells identified here have contacted epitope-bearing APC in the lung. If APC-CD4 T cell antigen dependent contact has occurred, it seems not to have resulted in detectable remodeling of CD4 T cell repertoire that is established in the dLN. Despite these limitations in our studies thus far, the data presented here provide new insight into tissue-specific homing after influenza infection and demonstrate that CD4 T cells of broad viral epitope specificity elicited after H1N1 influenza infection are recruited into the lung, where they then have the opportunity to encounter infected and/or antigen bearing APC and deliver effector function.

## Ethics Statement

This study was carried out in accordance with the recommendations of the University of Rochester Medical Center. All animal protocols used in this study adhere to the AAALAC International, the Animal Welfare Act, and the PHS Guide and were approved by the University of Rochester Committee on Animal Resources, Animal Welfare Assurance Number A3291-01. The protocol under which these studies were conducted was originally approved March 4, 2006 (protocol no 2006-030) and has been reviewed and re-approved every 36 months with the most recent review and approval January 23, 2018.

## Author Contributions

KR, AD, AR, and ZK helped design, perform and interpret experiments, HY performed statistical treatment of data, AS assisted in the design and interpretation of experiments. All authors assisted in the preparation of the manuscript.

## Conflict of Interest Statement

The authors declare that the research was conducted in the absence of any commercial or financial relationships that could be construed as a potential conflict of interest.
